# Mitigating amphibian disease: strategies to maintain wild populations and control chytridiomycosis

**DOI:** 10.1186/1742-9994-8-8

**Published:** 2011-04-18

**Authors:** Douglas C Woodhams, Jaime Bosch, Cheryl J Briggs, Scott Cashins, Leyla R Davis, Antje Lauer, Erin Muths, Robert Puschendorf, Benedikt R Schmidt, Brandon Sheafor, Jamie Voyles

**Affiliations:** 1 Institute of Evolutionary Biology and Environmental Studies, University of Zurich, Winterthurerstrasse 190, CH-8057 Zurich, Switzerland; 2 Smithsonian Tropical Research Institute, MRC 0580-12, Unit 9100 Box 0948, DPO AA 34002-9998, USA; 3 Departamento de Biología Evolutiva y Biodiversidad, Museo Nacional de Ciencias Naturales, CSIC, c/José Gutierrez Abascal 2, 28006 Madrid, Spain; 4 Department of Ecology, Evolution, and Marine Biology, University of California, Santa Barbara, Santa Barbara, CA 93106-9610, USA; 5 School of Marine and Tropical Biology, James Cook University, Townsville, QLD 4811, Australia; 6 School of Public Health, Tropical Medicine and Rehabilitation Sciences, Amphibian Disease Ecology Group, James Cook University, Townsville, QLD 4811, Australia; 7 Department of Biology, California State University at Bakersfield, Science 1/room 310, 9001 Stockdale Highway, Bakersfield, CA 93311, USA; 8 U.S. Geological Survey, Fort Collins Science Center, 2150 Centre Avenue, Bldg C, Fort Collins, CO 80526-8118, USA; 9 KARCH, Passage Maximilien-de-Meuron 6, 2000 Neuchâtel, Switzerland; 10 Department of Natural Sciences, Carroll College, 1601 North Benton Avenue, Helena, MT 59625, USA; 11 Department of Biological Sciences, University of Idaho, Moscow, Idaho 83844 USA

## Abstract

**Background:**

Rescuing amphibian diversity is an achievable conservation challenge. Disease mitigation is one essential component of population management. Here we assess existing disease mitigation strategies, some in early experimental stages, which focus on the globally emerging chytrid fungus *Batrachochytrium dendrobatidis*. We discuss the precedent for each strategy in systems ranging from agriculture to human medicine, and the outlook for each strategy in terms of research needs and long-term potential.

**Results:**

We find that the effects of exposure to *Batrachochytrium dendrobatidis *occur on a spectrum from transient commensal to lethal pathogen. Management priorities are divided between (1) halting pathogen spread and developing survival assurance colonies, and (2) prophylactic or remedial disease treatment. Epidemiological models of chytridiomycosis suggest that mitigation strategies can control disease without eliminating the pathogen. Ecological ethics guide wildlife disease research, but several ethical questions remain for managing disease in the field.

**Conclusions:**

Because sustainable conservation of amphibians in nature is dependent on long-term population persistence and co-evolution with potentially lethal pathogens, we suggest that disease mitigation not focus exclusively on the elimination or containment of the pathogen, or on the captive breeding of amphibian hosts. Rather, successful disease mitigation must be context specific with epidemiologically informed strategies to manage already infected populations by decreasing pathogenicity and host susceptibility. We propose population level treatments based on three steps: first, identify mechanisms of disease suppression; second, parameterize epizootiological models of disease and population dynamics for testing under semi-natural conditions; and third, begin a process of adaptive management in field trials with natural populations.

## Introduction

"The Amphibian Conservation Summit was called because it is morally irresponsible to document amphibian declines and extinctions without also designing and promoting a response to this global crisis." [1]

"Our focus on crisis has hampered conservation biology in achieving a scale of action required to match the world's environmental problems. Despite our best efforts to launch our cause into the mainstream culture, the world is suffering from crisis fatigue." [2]

**Figure 1:**
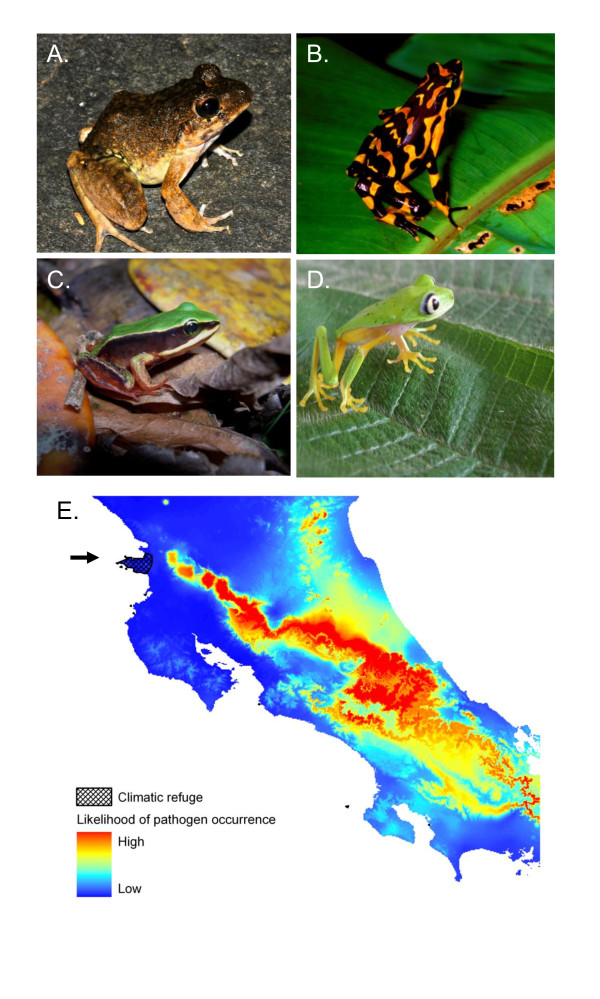
**Some of the rediscovered species in Costa Rica including**. A. *Craugastor ranoides*, B. *Atelopus varius*, C. *Lithobates vibicarius*, and D. *Pristimantis lemur*. Locality data are retained to discourage poaching. E. Climatic refuge in Costa Rica indicated by arrow. The core distribution of *Batrachochytrium dendrobatidis (Bd) *occurs in humid environments and coincides with the distribution of most declining populations of amphibians. Low abundance relict populations are being rediscovered within *Bd *enzootic zones, often with subclinical infections. Other species are found outside *Bd *enzootic zones. Healthy populations, in which a susceptible species maintained high abundance, were found at the edge of the distribution of the robber frog, *Craugastor ranoides*, in a climatic refuge [138]. Puschendorf *et al*. ([139]; including details of the bioclimatic model pictured in E) hypothesized that this relict population in the dry forest of Santa Elena Peninsula, Costa Rica, survives because climatic conditions in that habitat make pathogen establishment or persistence on hosts less likely.

Conservation biology is often characterized as a "doom and gloom" crisis discipline, a field of study that decries the loss of biodiversity and places blame on contributory human actions [3]. A prolonged sense of crisis and guilt with a continual focus on extinction is depressing. Such negative social perceptions of biodiversity conservation may exhaust public good will and become demotivating [4]. The effort to inspire and energize conservation biology can therefore benefit from fresh vision and the hope of restoration (Figure 1). Building on solid documentation and explanations for the loss of amphibians around the world, conservation research is now focusing on methods to halt and reverse this trend. Amphibian ecologists are entering a period of action in response to catastrophe [5].

Already, there are several successful amphibian conservation programs that have addressed problems of invasive species and habitat modification. For example, the removal of introduced trout from lakes in the Sierra Nevada mountains allowed recolonization of native frogs, reversing the effects of a major factor in population declines [6]. Another example is the restoration of wetlands and habitat corridors that clearly benefit amphibian populations in human altered landscapes [7,8]. One particularly unique program is to conserve the Kihansi spray toad (*Nectophrynoides asperginis*) from the Udzungwa Mountains, Tanzania. Large scale sprinkler systems were installed to compensate for water flow diverted by a hydroelectric dam. The managed habitat supported spray toads temporarily until a flood, and likely the disease chytridiomycosis, caused their extinction in the wild [9,10]. The demise of the Kihansi spray toad unfortunately demonstrates that factors causing population decline can act synergistically, often amplifying the effects of disease [11].

**Figure 2:**
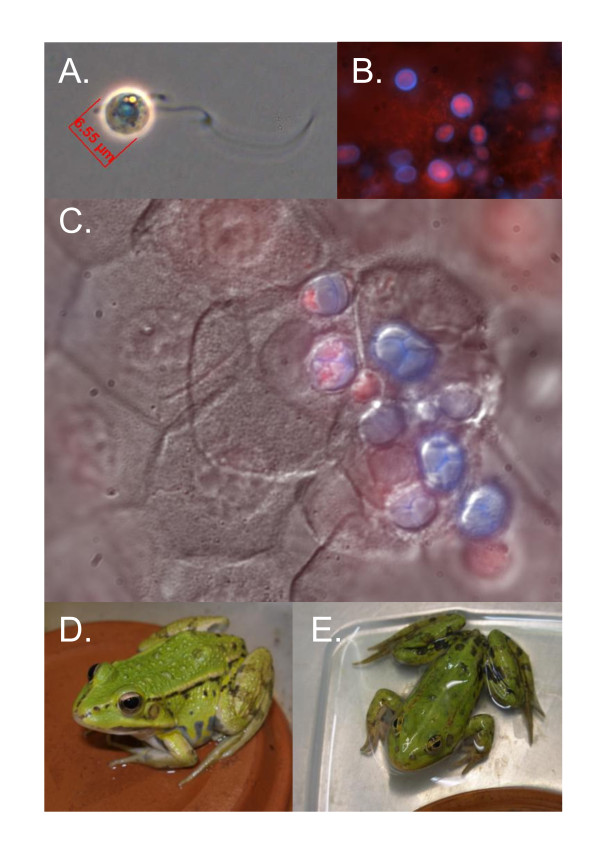
**Detection of *Batrachochytrium dendrobatidis *(*Bd*) and chytridiomycosis**. (A) infectious *Bd *zoospore 1000 × magnification stained with congo red. Infectious zoospores in the environment [20,229-231] or in association with amphibian skin may meet with host resistance mechanisms such as mucosal antibodies, antimicrobial skin peptides, or mutualistic bacteria [61,127,180,205]. (B) Small rod-shaped bacteria associated with sloughed skin from *Rana muscosa *are stained red with propidium iodide. Larger *Bd *zoosporangia are stained blue with calcofluor white. (C) *Bd *infecting frog skin 1000 × magnification stained with calcofluor white and propidium iodide. (D) A healthy infected frog, *Pelophylax lessonae*. (E) A diseased frog with chytridiomycosis. Note the skin shedding in water and splayed legs. If keratinized cells become infected, infections may be controlled by host responses that reduce *Bd *population growth resulting in host tolerance of low-intensity infection and no clinical signs of disease, as in (D). An alternative outcome of infection is uncontrolled *Bd *growth that leads to clinical chytridiomycosis (Figure 2E; reviewed in [207]). A simple definition of disease is uncontrolled infection.

Chytridiomycosis is caused by the fungus *Batrachochytrium dendrobatidis *(*Bd*), an emerging pathogen that colonizes amphibian skin [12,13] (Figure 2). This disease is a focus of many amphibian conservation efforts because of its nearly global distribution http://www.Bd-maps.net/ with recorded epizootics on several continents [1,14]. The impacts of chytridiomycosis differ radically among amphibian species and populations. Some are unaffected by *Bd *infection and act as carriers of the fungus [15,16] (e.g. bullfrogs, *Rana catesbeiana*). Some species tolerate a chronic, low level of infection, or experience a relatively slow population decline [17-19] (e.g. boreal toads, *Bufo boreas*) and some species experience severe, high levels of infection and acute population decline [20,21] (e.g. Panama poison dart frogs, *Colostethus panamensis*). There is evidence that these severe outbreaks can lead to the collapse of entire amphibian faunas including regional and global extinction [22-24] (e.g. Bob's robber frogs, *Craugastor punctariolus*).

Subsequent to disease emergence, natural recovery of populations is limited, regional amphibian diversity is homogenized [25], ecosystems are altered [26-29], and *Bd *becomes an established enzootic pathogen [30], often persisting in reservoir amphibian host species [16,17,31]. At these *Bd *enzootic locations, factors leading to lethal chytridiomycosis are not well understood, but local ecological context, particularly climate, is critical [18,32-36]. Although chytridiomycosis has rightly come to be regarded as "an alarming model system for disease-driven extinction..." [20], we rather view chytridiomycosis as an opportunity to test wildlife disease mitigation approaches and a model system to investigate disease dynamics in ecological systems.

## Aims of Disease Mitigation

Conservation priorities for amphibians threatened by chytridiomycosis are currently structured around preventing pathogen spread to unexposed populations, establishing *ex situ *assurance colonies, and developing *in situ *prophylactic treatment or remedial disease strategies [1,37-39]. No single solution is appropriate for all amphibian species with their rich diversity of life histories and habitats [40]. Here, we discuss seven population-level mitigation strategies against *Bd*. These strategies may have specific regional applications depending on social and environmental contexts. In most cases, local elimination of potentially harmful microorganisms is not practical because of the continued risk of pathogen reintroduction. We emphasize that elimination of *Bd *is not necessarily the desired management endpoint for the purposes of amphibian conservation because preventing disease does not always require eliminating exposure to pathogens. Furthermore, preventing population declines does not necessarily require eliminating disease.

The presence of a parasite or pathogen does not necessarily cause disease or amphibian population declines. There are many examples of serious pathogens that are "opportunistic" and normally present in healthy individuals. In amphibians, these include common environmental bacteria such as *Aeromonas*, *Flavobacterium*, *Pseudomonas*, *Acinetobacter*, and *Serratia *[41]. In humans, opportunistic fungal pathogens include *Candida albicans*, a commensal fungus found in the gastrointestinal tract of 40 - 60% of people, causing severe candidiasis only in immunocompromised patients [42,43]. Other medical examples are instructive [44-47], and demonstrate that a pathogen is not always pathogenic. Here we illustrate the opportunistic nature of *Bd *(Table 1, Figure 2), and suggest that the goal of wildlife management should be the preservation of viable, even if colonized, populations rather than the elimination of the pathogen.

**Table 1:** The ecology of *Batrachochytrium dendrobatidis *(*Bd*) opportunism indicates disease risk factors critical for focused management.

Pathogenicity	A review of the pathogenesis of *Bd *leads to the conclusion that the fungus is well adapted to the skin of amphibian hosts [228]. However, *Bd *can also be detected in the water column [218,229,230], and on moist substrates [20,231]. Although saprophytic growth is not strongly indicated, *Bd *forms biofilms in culture that could, hypothetically, improve survival under harsh or variable environmental conditions. Environmental longevity may entail life-history trade-offs that occur in response to culture conditions of temperature and nutrient availability [160,161], and specific host adaptations are not unlikely. *Bd *appears to exhibit chemotaxis toward favorable substrates [232], or away from unfavorable substrates (B. Lam & R.N. Harris, unpublished). Virulence of *Bd *appears to vary with the strain in laboratory experiments [49,68,69], although the determinants of differential virulence are not well understood [228].
Susceptibility	Susceptibility to amphibian chytrid occurs on a spectrum. *Bd *is an opportunistic pathogen that can be present transiently, cause sublethal host damage, or cause uncontrolled infections leading to death. Developmental stages of amphibians are not equally affected by exposure to *Bd*, and disease outbreaks of some species are associated with metamorphosis [206,233,234]. Figure 2 illustrates several outcomes of exposure to *Bd*. The contribution of immunopathology to host damage has not been characterized, however, at least in the cases of *Bufo bufo *and *Bufo boreas *tadpoles, immunopathology or physiological trade-offs can result from exposure to *Bd *even without infection [34,235] (Figure 3). Amphibian susceptibility is extremely sensitive to environmental context.

Environment	Outbreaks of chytridiomycosis are often the result of environmental forcing [36]. For example, although *Bd *can be widespread across a landscape, mid- to high-elevation populations are often more severely affected by disease than low-elevation populations of the same species. Seasonal disease dynamics are another manifestation of environmental context-dependency, and high infection prevalence is often associated with cool seasons (reviewed in [207]). Climatic variability is also associated with epizootic disease dynamics [67]. A growing number of studies demonstrates that exposure to *Bd *does not always cause infection, and many species and regions appear to be unaffected by disease [64,207,236] (Figure 1). This begs the crucial question for disease management: What factors lead to host protection?

Within the spectrum from transient commensal to acute lethal disease, the sublethal effects of *Bd *exposure may be considered as mild controlled infections or as latent effects (originating from an earlier exposure but expressed in hosts without infection after a period of clinical quiescence [48]; Table 1, Figure 2). Controlled infections or even transient exposure to *Bd *may cause a reduction in mass or growth rate [34,49-51] or other consequences that reduce host fitness. Since these effects are not due to an uncontrolled infection, the animals do not have chytridiomycosis by our simple definition (Figure 2). Rather than aclinical chytridiomycosis, measurable clinical signs with no detectable *Bd *colonization may be the result of an effective, yet costly, immune defense.

**Figure 3:**
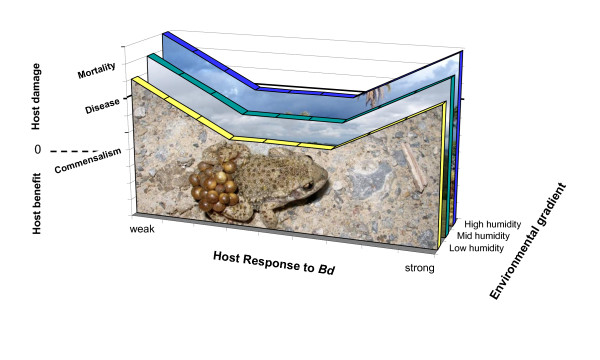
**A conceptual model, developed here, for an ecological damage-response framework of *B. dendrobatidis (Bd) *pathogenicity**. At either extreme of host response the host damage (disease) progresses toward mortality. At mid-levels of host response to *Bd *there may be no host damage (subclinical infection), depending on the environmental context. Environmental gradients may include elevation, temperature, temperature variability, pesticide concentration, intensity of co-infection, or other factors. Shown here is a reduced damage-response curve at low environmental humidity. Theoretically, chytridiomycosis is suppressed where environmental conditions are not conducive to *Bd*, but under some environmental conditions host defenses become critical for control of chytridiomycosis. Other damage-response curve shapes are possible for this opportunistic pathogen.

An alternative framework defines the disease chytridiomycosis in terms of host damage caused by *Bd*, irrespective of *Bd *infection status. That is, microbial pathogenesis is the outcome of microbe, host, and environmental contributions and interactions (Figure 3). This ecologically-oriented damage-response framework [52] takes into consideration that pathogen strain, environmental conditions, and host behavior, genetics, and immunity can affect the disease (or damage) response. This concept incorporates a threshold burden of *Bd *infection that leads to death [17,21,53], and the potential for immunopathology (damage caused by the host immune response rather than the pathogen) that can lead to host damage irrespective of infection intensity [34,52,54,55]. In Figure 3, we model the relatively greater damage caused by *Bd *when host responses are too weak or too strong, and how this response can differ across environmental gradients. This ecological immunology framework is ideal for chytridiomycosis mitigation because some strategies to reduce disease focus on environmental context or host behavior/immunology, rather than on limiting the pathogen.

Increasing numbers of studies show that some amphibians can clear *Bd *infections (Table 2), and in at least one study, can reduce infection loads [56]. This can be dependent on environmental conditions and host behavior [19,57], life-history strategy [34], or an immune reaction that suppresses infection intensity after a peak between 7 - 40 days after exposure [57-61]. Catastrophic mortalities may be less common once the pathogen is an established enzootic. Although severe chytridiomycosis and amphibian mortality, as with many wildlife diseases [62], is notoriously difficult to detect in the field, severe chytridiomycosis is rarely observed in many high *Bd *prevalence amphibian populations [17,18,57,63,64].

**Table 2:** Studies that show clearance of *Batrachochytrium dendrobatidis *(*Bd*) from infected amphibian hosts.

Reference	Amphibian species	Lab or field	Temperature
Woodhams *et al*. [90]	*Litoria chloris*	Lab	2 d × 8 hr at 37°C
Berger *et al*. [58]	*Mixophyes fasciolatus*	Lab	Constant 27°C
Retallick and Miera [49]	*Pseudacris triseriata*	Lab	5 d at 32°C
Bishop *et al*. [59]	*Leiopelma archeyi*	Lab	Constant 15°C
Becker and Harris [51]	*Plethodon cinereus*	Lab	Constant 17°C
Kriger and Hero [237]	*Litoria wilcoxii*	Field	Natural
Corn [238]	*Bufo boreas*	Field	Natural
Murray *et al*. [147]	*Litoria pearsoniana*	Field	Natural
Pilliod *et al*. [19]	*Bufo boreas*	Field	Natural
Briggs *et al*. [17]	*Rana muscosa*	Field	Natural
Voordouw *et al*. [239]	*Rana pipiens*	Field	Natural
Geiger *et al*. [91]	*Alytes obstetricans*	Lab	5 d at 28°C
Márquez *et al*. [240]	*Hypsiboas crepitans*	Lab	Constant 23°C
Chatfield & Richards-Zawacki [92]	*Rana catesbeiana *and *Acris crepitans*	Lab	10 d at 30°C

Clearance of *Bd *from marked individuals in field studies may have been caused by fluctuations in seasonal environmental conditions, but other factors are possible.

Disease management in these seemingly commensal cases or enzootic areas remains important for several reasons. First, population growth or amphibian abundance may be suppressed by enzootic *Bd *[18,19,57]. Second, *Bd *exposure and infection has sublethal costs [50,65,66]. Third, changing environmental conditions [67] or more virulent strains [49,68,69] may disrupt the temporarily commensal relationship between amphibian host and *Bd*, as with other opportunistic pathogens. Thus, the experimental disease mitigation strategies described below are designed for both pathogen naïve populations and persistently infected populations with a view toward epidemiological modeling and adaptive management of an opportunistic pathogen.

## Experimental disease mitigation strategies

**Figure 4:**
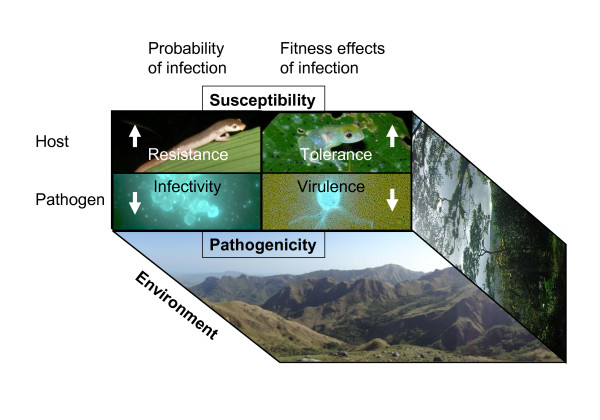
**Four categories of host-pathogen dynamics**. Arrows indicate the desired direction for an effective disease mitigation strategy. Here infection and the effects of infection are distinguished, and the host-pathogen interaction is placed within an environmental context. The epidemiological triad of environment, host, and pathogen produces complex interactions affecting health and disease such that each of the dynamics and the disease outcome may vary in different environments. In general, disease is a product of host susceptibility and parasite pathogenicity in a given environment. Susceptibility and pathogenicity each have two basic components relating to the probability of infection and the host fitness effects of infection. Host susceptibility is described by the relative resistance to becoming infected, and the relative tolerance of the host (controlling disease development [123]). Tolerance can be described "as the ability to limit the health or fitness consequences of *a given parasite burden*" and can be statistically quantified [241]. Likewise, parasite pathogenicity is described by the relative ability to infect a host (infectivity), and the relative severity of disease (virulence). Some studies predict the fixation of tolerance genes in affected populations, and the maintenance of polymorphism in resistance [123,242]. Disease control strategies can manage for levels of resistance, tolerance, infectivity, or virulence. Here, transmission is considered a component of infectivity.

Because it is too early for a review of experimental results, this section is intended to focus on the conceptual stages of designing effective population-level disease management strategies. Each section below refers to similar management practices for human, veterinary, or wildlife diseases, discusses the mechanism of the strategy (Figure 4: resistance, tolerance, infectivity, virulence), and presents an outlook on the potential usefulness of the strategy for amphibians.

## Reducing host density to prevent disease outbreaks

Reducing the density of susceptible hosts in a population by culling or translocating individuals may limit pathogen transmission and infectious doses, thus reducing the risk of disease outbreak [70,71]. Culling of livestock is often used to prevent economically destructive and zoonotic disease outbreaks such as foot and mouth disease (virus [72]), pandemic influenza (H5N1 influenza A virus [73]), or Johne's disease (*Mycobacterium avium *subsp. *paratuberculosis *[74]). Reducing host densities in wildlife is also common for large mammals such as African elephants, American bison and elk, or kangaroos, feral horses and camels in Australia. Though culling can be controversial [75] or ineffective (e.g. for suppression of Tasmanian devil facial tumor disease [76], or for suppression of white-nose syndrome in bats [77]), this approach may be particularly useful for managing spatial distribution and connectivity within host metapopulations and was used successfully against rinderpest [78]. Since field experiments suggested that transmission of *Bd *may be host density dependent [79], reducing the susceptible host population at a predicted outbreak site may reduce disease risk. In one trial, *Rana muscosa *densities were reduced by translocating uninfected hosts to habitats that formerly sustained populations, rather than culling. These preliminary experiments in the California Sierra Nevada have not been particularly successful at preventing outbreaks or restoring populations (V.T. Vredenburg, R.A. Knapp pers. comm.).

## Treating amphibian hosts and habitats

Limiting the prevalence of infection or the infectious dose accessible in the environment by treating individual hosts or habitats may reduce pathogenicity and prevent disease outbreak. Perhaps one of the oldest and most common strategies against fungal diseases is agricultural fungicides [80,81]. Antibiotics [82] and salt [83] against fungal diseases are commonly used in aquaculture and for veterinary treatments of fish and amphibians [84]. Besides chemical treatments, management including drainage of entire wetland systems is not unordinary for control of mosquito vectors of human diseases [85,86].

**Figure 5:**
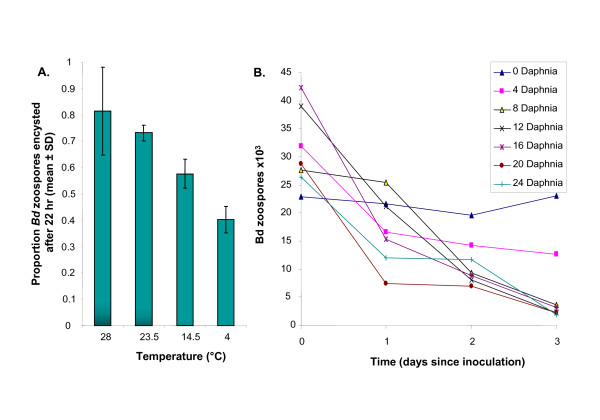
**Abiotic and biotic factors affecting *B. dendrobatidis(Bd)* zoospores.** A) *Bd *zoospore attachment and encystment varies with temperature in culture and is thought to alter infectivity [160]. B) The biotic environment can also alter *Bd *abundance. Shown here is the effect of inoculated population size of grazing *Daphnia *on the quantity of *Bd *zoospores over three days (A. Lauer, see Appendix for methods).

*In vitro*, *Bd *is susceptible to drying, salt, and a broad range of antibiotics and chemicals [87]. Longevity of zoospores is dependent on temperature (Figure 5A), and *Bd *in sterile lake water can remain infective for up to seven weeks in state of slowed development, rather than saprophytic growth, until conditions improve [88]. Competing microbes and predators can also reduce longevity of the pathogen [89] (Figure 5B). Infected hosts have been successfully treated with heat [49,90-92] or antibiotic applications [93,94].

Mathematical models suggest that both individual host-level and habitat-level treatments against *Bd *may be effective management tools. The Briggs *et al*. [17,95] models suggest that amphibian populations can recover or persist in low abundance if some individuals lose infection. The mathematical model developed by Mitchell *et al*. [96] predicts that the longer the fungus is able to survive in the water, the greater the impact on host populations, suggesting that free-living stages of the pathogen should also be targeted.

At present, we are unable to completely eliminate *Bd *from an amphibian population or community because we lack essential information about where *Bd *occurs in the environment and how it spreads. Therefore, any attempts to reduce the levels of *Bd *in an amphibian population should be focused where success is most likely: (i) where we have access to a considerable proportion of the *Bd *population including infected hosts, (ii) where the amphibian community is relatively simple and/or (iii) where the habitat is relatively simple or isolated such that treatments can be controlled (e.g. a clearly delimited pond). Pilot treatment regimes in mesocosms (C. Geiger, pers. comm.) and in natural populations are making progress in Australia (M. Stockwell, pers. comm.), California (V.T. Vredenburg and R. Knapp, pers. comm.), and in Spain [5]. Treatments include antifungal chemicals, salinity, and pond drying to suppress *Bd*. Details of several ongoing studies can be found in the appendix.

The characteristics that restrict *Bd *indicate potential methods to manage *Bd*, such as creating areas of shallow water, canopy-free zones [97], or heating stations in managed wetlands to increase host thermoregulatory opportunities. Ecosystem engineering by beavers may have similar effects on water temperature and can benefit amphibian communities [98,99]. Re-creation of these historically natural processes by human managers must be done with care to avoid disrupting amphibian distributions and by testing whether hosts are behaviorally and phenotypically equipped to take advantage of such measures [100]. Reducing *Bd *infection prevalence or the probability of transmission to a level where amphibian populations can coexist with the pathogen may require repeated treatments; in many areas, continuing habitat management is necessary to maintain viable amphibian populations [40,101]. The conservation biologist's toolbox is becoming equipped for managing amphibians and habitats with periodic physical or chemical treatments.

## Reintroduction with assisted selection

Returning animals to the wild after extirpation is often an attractive option for managers, especially in "pristine" localities where the native fauna is protected and maintenance of the existing flora and fauna is mandated (e.g. national parks and nature reserves). Such programs include repatriation of rescued wildlife, translocation of wildlife from a more prolific region, or reintroduction of offspring that have been raised in captivity.

Reintroductions of amphibians have had mixed success [101-104]. The programs can be expensive and labor-intensive, and complicated by potential adaptation to captivity, and the presence of disease in the captive population or at the release site [104-106]. Many of these challenges, however, can be addressed. For example, to prevent genetic adaptation to captivity, breeding programs can minimize the number of generations produced before release, delay reproduction, or cryopreserve eggs and sperm if release is not imminent [107,108]. When properly executed and monitored [103,109,110] reintroductions have potential for success (e.g. the natterjack toad, *Bufo calamita *reintroduction in the U.K.[101]). Artificial selection has been successful to improve resistance to viral and bacterial pathogens in livestock [111,112] and in many fish species, *i.e*. [113-115]. Incorporating disease resistance into amphibian reintroduction programs may be desirable for species threatened by chytridiomycosis.

Similar to fish, high fecundity and short generation times of many amphibians may make them well-suited to selective challenge with *Bd*, using survivors as breeding stock for the next generation. Many amphibian species, however, produce very few eggs or their captive husbandry remains obscure. In these cases, a possible alternative to selection by pathogen exposure is to select for specific, measurable immunological characters that have the potential to impart resistance. Gaining an in-depth understanding of amphibian immunity is critical.

A strong candidate for this type of experiment would be selection for effective antimicrobial peptides (AMPs; [116]). Large quantities of AMPs are produced in the skin granular glands of many amphibians as an investment in the innate immune system. The ability of amphibian AMPs to inhibit *Bd *growth *in vitro *has been shown to positively correlate with resistance to chytridiomycosis [117] and has been used to predict disease susceptibility among species and populations [118,119]. Because AMPs can be collected by noninvasive techniques and the amount and effectiveness of the peptides produced by each individual can be assessed [120-122], developing a screening process for individuals with the most effective peptide repertoires has potential for use with selective breeding.

This approach hinges on whether enhanced AMP expression reduces susceptibility to chytridiomycosis and whether the effectiveness or quantity of AMPs produced among individuals is variable and heritable. Evidence is mounting to demonstrate these prerequisites: An increase in *Bd *infection intensity resulted from reducing AMPs in young African clawed frogs, *Xenopus laevis *[61]. AMP production changes little upon entry into captivity [119], AMP expression is induced upon pathogen exposure in some disease resistant species (D.C. Woodhams, unpublished), and AMP expression is both heritable and variable among individuals [119,122]. Immune defense genes such as those encoding AMPs that allow for tolerance of *Bd *may be rapidly fixed in a population [123,124]; whereas, the frequency of genes allowing resistance to infection may fluctuate [116]. Although AMPs may have a role in both tolerance and resistance, some species such as Panamanian golden frogs, *Atelopus zeteki*, and boreal toads, *Bufo boreas*, among others, do not appear to produce anti-*Bd *skin peptides [125,126]. Other heritable defenses including both innate and adaptive defenses [61,127,128] may be better targets in these species, and such defenses may be best identified in remnant populations (Figure 1).

In some cases, reintroduction programs can also benefit from natural selection for disease resistance by focusing on populations that have persisted beyond initial outbreaks of chytridiomycosis. From such a population at Peñalara Natural Park in Spain, founders from relict metapopulations of midwife toads, *Alytes obstetricans*, were captured after 10 years of successive and severe mass mortality events. Natural selection has been shown to occur even in such short time frames (e.g. [129]) and surviving toads appear less susceptible to disease (J. Bosch, unpublished). Similarly, in the Rocky Montains, USA, some populations of boreal toad, *Bufo boreas*, persist with disease [19]; the mechanism is unknown, but some genetic lines have survival advantages [57]. By attenuating disease-induced population declines long enough for natural selection to produce disease resistance, captive colonies and the problems associated with artificial selection can be avoided. This strategy is being employed in Australia for the critically endangered Corroboree Frog, *Pseudophryne corroboree*. Field-collected egg masses are raised in predator-free mesocosms to head-start populations [130]. Preserving the full range of amphibian habitats is essential for this strategy because environmental conditions that allow hosts an advantage over disease may occur in only a subset of habitats.

## Climatic refugia and the management implications of species rediscovery

Discovery and rediscovery boost the public perception of conservation, often arousing imagination and hope through positive media coverage [131]. Some rediscoveries are controversial, such as sightings of ivory-billed woodpeckers in the Big Woods of Arkansas [132-134]. These rediscoveries have influenced the development of guidelines for conservation listings [135] and criteria for designating an organism as "extinct in the wild" [136]. Such designations have significant management implications.

Rather than obscure sightings or calls, several species of amphibians have been captured after long absences, making media headlines (Figure 1A). For example, a sister-species to the famous and extinct golden toad, Holdridge's toad, *Incilius holdridgei*, was also thought to be extinct for 25 years until rediscovered in a *Bd*-enzootic region of Costa Rica that formerly supported an abundant population [137]. Most such rediscoveries are quite recent, giving hope that more amphibian species thought to be extinct may persist in the wild in a relict population, perhaps in climatic refugia.

Climatic refugia may arise under two different situations, (1) areas where susceptible host species persist in association with pathogens, or (2) areas where susceptible hosts persist outside the distribution of the pathogen (Figure 1). Environmental conditions may suppress disease development by decreasing pathogenicity (inhibiting pathogen growth or transmission), decreasing susceptibility (allowing effective host responses), or both. These refugia, therefore, represent areas of high conservation value since they may harbor source populations. Species distribution models may help to identify climatic refugia [138-141] (Figure 1).

As an example of the first situation, after the introduction of avian malaria into Hawaii, several species of birds persisted in high abundance in upland xeric habitats where breeding capacity of mosquito vectors is limited in comparison with lowland mesic habitats where bird populations crashed in association with disease [142]. Since then, some species of endemic birds have recovered, despite prevalent low-intensity chronic infections, suggesting that at least Hawaii amakihi, *Hemignathus virens*, have evolved tolerance to the pathogen under climatic conditions favorable to disease transmission [143,144]. Remnant populations of disease resistant birds in the lowlands may have been critical for recovery [144].

A similar phenomenon may be occurring in some amphibian populations. Many species with large ranges across altitudinal gradients have declined to the point of local extinction at upland sites but persisted in coexistence with *Bd *at lowland sites [145,146]. Although mortality still occurs at lower elevations, particularly when environmental conditions are most conducive for disease development [147], lowland populations of susceptible species of frogs persist with infection. For example, lowland frogs on the east coast of Australia have persisted with infection for at least 15-20 years since the initial outbreaks of chytridiomycosis [30,147]. Populations of some susceptible frog species have recovered and have begun to re-colonize upland sites from which they were extirpated during initial outbreaks of chytridiomycosis [10]. Because of ongoing exposure to *Bd*, selection on these frogs may have altered behavioral patterns or enhanced immune functions such as antimicrobial peptide defenses [122], symbiotic microbial communities, or adaptive immune defenses. If relict populations successfully coexisting with *Bd *maintain enhanced capacities for infection resistance or tolerance compared to pre-decline populations, they could provide the genetic resources for breeding resistance in survival assurance colonies.

In amphibian populations that persist with *Bd *infections, the balance for host-pathogen coexistence may be tenuous and these populations remain highly threatened for several reasons. To begin with, relict populations that survive initial declines often persist with dramatically reduced abundances and are therefore vulnerable to stochastic processes. If these populations persist in environments that are favorable for *Bd *growth (e.g., mid to high elevation rainforests), *Bd *can have prolonged detrimental effects on recovering populations [18,19,147,148] that prevent the return to pre-decline abundances. Additionally, these populations remain threatened by the introduction of new and potentially more virulent strains of *Bd *and their risk of suffering disease-induced extinctions could be exacerbated by environmental change (even in areas that are currently considered climatically-protected areas [67,149]). Thus, continued management of core habitat and adjacent areas will remain essential if these depressed populations are to persist and recover in the long run.

The current distribution of some species such as *Craugastor ranoides *has developed in response to disease pressure. Within this group of closely related frogs most species are extremely susceptible to chytridiomycosis and have gone through significant declines, and many are thought to be extinct [20,23,138,150]. The high abundance of *C. ranoides *in the dry forest of Costa Rica is an example of a second type of climatic refuge that arises when there is little overlap between the distribution of the host and the pathogen (Figure 1). Identification of this type of climatic refuge is important for potential managed relocation similar to that proposed to combat biodiversity loss due to climate change [151].

For species presumed to be extinct in the wild, continued monitoring of historical sites and exploration of adjacent areas remains an important task even after significant periods with no sightings. Habitat protection is crucial because without it, future rediscoveries of "extinct" species might not be possible. Relict populations can also provide important insights; understanding how they escaped pathogen exposure or survived the initial outbreaks of *Bd*, and how they persist, despite the presence of this pathogen, is key to developing effective management strategies.

## Immunization to fortify amphibians and attenuating *Bd *for a live vaccine

Immunizing amphibians is perhaps one of the most intuitive disease mitigation strategies. Immunization is common in human and veterinary medicine; it has been employed in efforts to eradicate rinderpest and foot and mouth disease in ruminants [152], and against sylvatic plague in black-footed ferrets [153]. In wild populations this strategy works through herd immunity: producing a threshold proportion of hosts that are resistant to infection in order to suppress disease outbreaks in sensitive populations [154,155].

Immunization can be achieved through a variety of processes that fortify amphibian immune systems against *Bd*, either suppressing disease development or preventing an infection. There are multiple lines of evidence that suggest that an immunization strategy could be successful either by attenuating *Bd *pathogenicity for use as a live vaccine or by strengthening amphibian resilience to pathogen exposure.

First, *Bd *virulence is known to vary among strains [49,68,69]. The rapid range expansion of *Bd *may indicate the emergence of a highly virulent strain that now predominates [156]. Molecular studies indicate that genetic differences exist among strains [69], and further research may identify those that are inherently less virulent to susceptible amphibians [157]. Discovery or development of an attenuated strain will hinge on resolving the mechanisms that result in hyper or hypovirulence. Promising insights into these processes are emerging: A threshold dose needed for infection and disease development is one virulence mechanism common to many pathogens [158] including *Bd *[17,53]. In fact, this "group effect" is even apparent in isolation and culture of *Bd *such that single zoospores or small clusters of sporangia do not readily continue development [159]. Plasticity in *Bd *life-history characteristics under different environmental conditions may correlate with virulence [160,161]. Stable *Bd *life-history adjustments also occur in response to culture conditions of temperature and nutrient availability [161]. Ongoing artificial selection experiments will determine whether *Bd *virulence is amenable to changes in culture. In addition to dose effect and life-history characteristics, virulence can be attenuated in culture by heat or chemical treatment, or by genetic modification of some fungal pathogens [162,163]. A low virulence stain of *Bd *may not have a competitive advantage among strains unless it is first to colonize the host and it stimulates host immunity that prevents further infections.

Second, an untested hypothesis is that frogs normally susceptible to chytridiomycosis, once cleared of infection by manipulating environmental conditions or by chemical treatment [49,90,93,94], will have enhanced immunity upon re-exposure to *Bd*. Murphy et al. [57] showed that boreal toads, *Bufo boreas*, can recover from a low dose of *Bd*, however it is unknown whether recovered individuals are more resistant to disease development upon subsequent exposure. Amphibians can develop an adaptive immune response against killed *Bd *injected directly, but this response has not reduced susceptibility to infection in the species studied (*Bufo boreas *[164] and *Rana muscosa *[127]). In a recent study of disease resistant African clawed frogs, *Xenopus laevis*, antibodies were found in the skin mucous layer that bind to *Bd *[61]. Future research directed at immunization protocols to ramp up mucosal, rather than circulating, antibodies in disease-susceptible amphibian species may be fruitful, particularly to safeguard captive animals, although the method lacks the potential to protect future generations of non-immunized amphibians.

## Habitat bioaugmentation and host biotherapy

Protective microbiota are potentially disrupted by increasing environmental changes (e.g. habitat alteration, climate change) equivalent to suppressing innate immune defenses [165-167]. Thus, the microbiota associated with an amphibian host or species may not be capable of inhibiting *Bd*. To increase this capacity, bioaugmentation or biotherapy is a strategy to add a beneficial (probiotic) strain or consortium of microbiota to amphibians or to their habitat for the purpose of reducing host susceptibility to infection or disease [168,169]. Usually the microorganisms applied in this strategy are already or were historically present in the habitat, rather than introducing new organisms to an already stressed system. Biostimulation is a similar strategy of adding nutrients or compounds (prebiotics) to promote the growth of beneficial microbiota relative to potential pathogens [170].

There is a long-standing practice of repeated bioaugmentation applications, including fungi, to prevent disease in agriculture [171,172]. Bioaugmentation with another fungus or a hypovirulent strain of the same pathogen may be useful to prime the host's immune system as in the semi-successful treatment of chestnut blight (*Cryphonectrua parasitica*) in the U.S. by treatment with an innocuous European strain [173]. Increasingly, bioaugmentation is practiced in aquaculture and is under development for ecosystem-wide restoration of coral reefs [174]. In the later case, native coral-associated microbial communities are capable of excluding pathogens, producing biocides, and interfering with pathogen cell signaling. There is mounting evidence for a similar function of amphibian skin microbiota at maintaining host health; bacteria such as *Pseudomonas *spp. and *Janthinobacter *spp. are capable of suppressing disease in some amphibians [50,51,89,175-180]. Similar to human gut microbiota, a specific microbial consortium associates with amphibian skin and may regulate host immunity ([181,182], L.R.D., E. Küpfer, and D.C.W., unpublished data).

Restoring or enriching commensal microorganisms in amphibian populations will involve studies on the diversity of microbial consortia present in amphibian skin and their environment, persistence of the microbial communities over time, and modes of transmission. Current laboratory trials are underway to assess *Bd *inhibitory activity of symbiotic microbes and to test for resistance to host antimicrobial peptides and other defenses. Studies are progressing from the laboratory to mesocosms, and in at least one case, to an emergency bioaugmentation application in controlled field studies for the critically endangered mountain yellow-legged frog, *Rana muscosa*, in the Sierra Nevada, California (R.N. Harris, pers. comm.; [183]).

A related strategy is to look for pathogens that specialize on *Bd *and to apply these to infected frogs or habitats. For example, pathogenicity of *Cryphonectria parasitica*, the fungal agent of chestnut blight can be reduced by mycoviruses. At least ten families of mycoviruses have been detected; their effects range from decreasing to increasing the fitness and pathogenicity of their fungal hosts. These viruses can be directly useful for biocontrol of fungal pests on plants and have potential for use as gene vectors to modify fungal virulence [184]. Mycoviruses of *Bd *have not yet been detected.

## Biocontrol with predators of *Bd*

In addition to microbial competitors, *Bd *has natural predators that could be used as biocontrol for disease. In particular, microcrustacean zooplankton, such as water fleas (Cladocera), copepods (Copepoda), and seed shrimp (Ostracoda), are aquatic grazers and eat the aquatic zoospores of some chytrid fungi [185]. Based on this observation, zooplankton may be important ecological regulators of *Bd *populations and reduce the risk of amphibian infection in aquatic environments. Copepods are successfully used as biological control agents in other disease systems; for example, applications of *Mesocyclops *reduce populations of mosquito larvae suppressing the vectors of Dengue Hemorrhagic Fever and reducing disease incidence [186].

The efficacy of the biocontrol approach for chytridiomycosis will depend on clarifying the ecological interactions of microcrustaceans in pond systems and testing the hypothesis that microcrustaceans can influence *Bd *population densities. Preliminary studies have found that microcrustaceans reduce *Bd *zoospore densities in laboratory culture experiments (Figure 5B). Isolated microcrustaceans (cladocera - *Daphnia sp*. and ostracods - *Cypridopsis adusta*), which are common in freshwater of ponds of the foothills of the Sierra Nevada and the Southern San Joaquin Valley [187], subsisted on only *Bd *zoospores for seven days, while microcrustaceans in cultures with no *Bd *zoospores died. *Bd *populations were significantly reduced in these cultures. Even small densities of *Daphnia *reduced zoospore populations significantly (Figure 5B). However, it is not known if microcrustaceans feed on zoospores when other food is available. Future experiments will test zooplankton grazing efficiency in mesocosms with alternative food sources.

One hypothesis that links microcrustacean predation with the ecology of *Bd *is that changes in microcrustacean diversity could alter the population dynamics of *Bd *in the water column and thereby influence disease dynamics in amphibians. Diversity of microcrustaceans appears to be declining in alpine lakes in California [188-190]. Microcrustaceans are important components of aquatic food webs worldwide; they are sensitive to environmental changes and therefore excellent indicator taxa for pollution (e.g. [191-193]), climate change and introduced species [188]. Ongoing studies will characterize both the seasonal microcrustacean diversity in the water column, and the historical diversity by examining eggs surviving for up to several hundred years in the sediment [194]. If the loss of microcrustacean diversity is important to the disease dynamics of chytridiomycosis, ponds with degraded communities that are *Bd *positive could be targeted for microcrustacean restoration management.

## Other strategies

**Figure 6:**
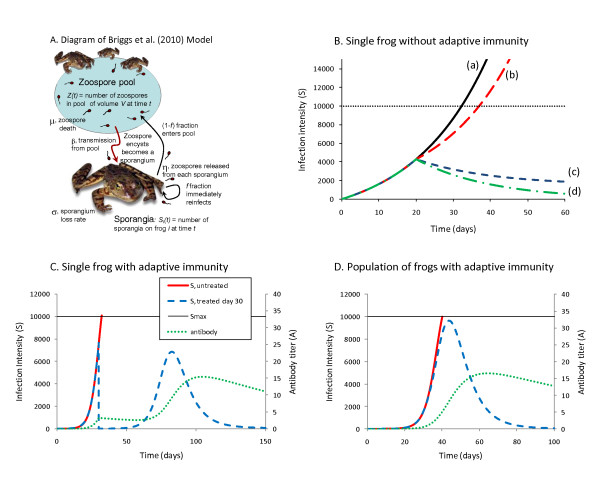
**Models of *B. dendrobatidis (Bd)* infection altered by management**. (A) Diagram of the Briggs *et al*. [17] model. The model follows the dynamics of the zoospores (Z) in a zoospore pool, and the sporangia (S_i_) on each frog i. Copied with permission from Proceedings of the National Academy of Sciences. (B) Examples of conservation strategies without an adaptive immune response: (a) frog remains untreated, (b) zoospore pool is eliminated on day 20, (c) constitutive defenses of the frog are increased on day 20, perhaps through the application of probiotic bacteria, and (d) both types of treatments are applied. Model details can be found in the appendix. (C) Examples of a treatment strategy if the frog has an effective adaptive immune response. (D) At the population level, reducing the density of frogs can slow the rate of increase of *Bd *in the zoospore pool, and give the frogs extra time to mount an effective immune response. In the untreated population of 100 frogs, the frogs' average fungal load (solid red line) increases rapidly to S_max_, and the population goes extinct. In the treated population, the frog density is reduced to 10 frogs on day 30. The rates of increase of *Bd *both in the zoospore pool and on the frogs (dashed blue line = average S per frog) are decreased, and the frogs' immune response (dotted green line = average antibody level per frog) is able to suppress the infection before S_max _is reached.

Disease mitigation strategies are not limited to those discussed here. It is worth pointing out that ultimately, broader conservation practices aimed at minimizing habitat modification, invasive species, atmospheric change, and environmental pollutants will reduce the need for downstream disease mitigation [195]. Meanwhile, integration of empirical studies and quantitative modeling (Figure 6) enables strategic planning of management solutions.

## Epizootiological models incorporating disease control strategies

Epizootiological models can elucidate the dynamic processes of infection, disease, and recovery at the individual host level, and disease-induced fluctuations at the population level. Disease control strategies can be incorporated into these models and simulations can be run to test different scenarios. Figure 6A shows that the growth rates of *Bd *can be manipulated by temperature. Strategies that act to reduce the number of infectious zoospores or slow the growth rate of the pathogen can be combined with data on transmission efficiency and rates of host immune responses. For example, Figure 6B shows that slowing *Bd *growth or reducing zoospores may allow time for host immune responses to reduce infection burdens. Scaling up to the population level, prophylactic treatments of naïve populations, or treatments of populations with enzootic *Bd *may allow amphibian population persistence without elimination of infection or all cases of disease. In this scenario, re-introductions of extirpated amphibians can begin before eliminating risk of *Bd *infection. These scenarios were modeled for *Rana muscosa *populations that either go extinct or persist with low infection burdens but high prevalence [180], and indicate that persistence is possible if some adults can survive infection and reproduce [95]. Additional context-specific modeling can help to assess hypotheses prior to field implementation of mitigation techniques. The reproductive success of additional species may depend on herd immunity thresholds.

Lam *et al*. [196] suggest a herd immunity threshold of approximately 80% in *Rana muscosa *populations exposed to *Bd*. If at least 80% of frogs are protected from disease by anti-*Bd *microbial symbionts, then the population can persist following disease emergence or introduction. This estimate provides several epizootiological insights. For example, the basic reproductive rate (R_0_), or number of new infections arising from an infected frog arriving at a pond, a measure of parasite fitness, must be less than 5 in a basic SI model where proportion protected, p > 1 - (1/R_0_). In a population estimated at 200 individuals, and given density dependent transmission, the maximum transmission efficiency (β) would be no greater than 5/200 = 0.025 new infections per day. If correct, experimentally reducing transmission efficiency below this threshold should prevent host decline in response to disease emergence.

## Ecological Ethics of Amphibian Disease Management

**Figure 7:**
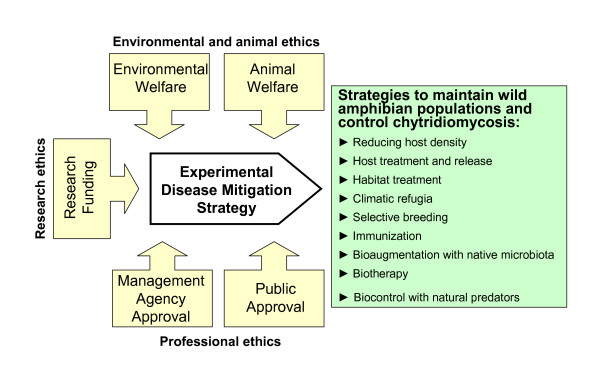
**Ecological ethics framework **[197,198]** supporting experimental disease mitigation strategies aiming to reduce *Bd *pathogenicity or amphibian host susceptibility and to allow long-term population persistence and co-evolution with a potentially lethal pathogen**.

Ecologists and conservationists working on disease mitigation experiments bear the ethical burdens both to act urgently on behalf of threatened biodiversity and to avoid excessive ecological risk or animal suffering. These responsibilities can be balanced within an ecological ethics framework [197,198] which blends guidance from multiple stakeholders, effectively diffusing the ethical burden on the experimenter. Consideration for animal welfare, the welfare of the environment, the concerns of funding agencies, parks and wildlife agencies, and public perception merge on an ethical course of action (Figure 7). An appropriate ecological ethics framework garners respect for the practice of conservation biology and enthusiasm for experimental disease mitigation projects without lingering moral fears. Approval and permitting systems are in place in many countries that provide these services to scientists, but continued bioethical thought is needed for emerging questions:

► What is the best management practice for rediscovered populations consisting of very few individuals?

► What level of risk is warranted to reduce the untold suffering of wild amphibians succumbing to disease and the destructive downstream ecosystem cascades?

► What are the critical biotic components that must be considered before using treatments aimed at saving amphibian populations from catastrophic decline?

► What scale of application is appropriate for disease mitigation strategies that alter natural habitats, biotic communities, or host genotypes?

► Are live vaccines or genetically altered pathogens ethically viable options for wildlife disease mitigation?

Ethical science, public participation, education, and the political-values struggle are intertwined with conservation efforts in general [199,200]. Grass-roots support is critical to facilitate action through media campaigns and fund raising once the best conservation strategies are determined, and this is growing thanks to groups like Save the Frogs, Amphibian Ark, World Association of Zoos and Aquariums, and others [201,202]. With this firm footing, we can meet the challenge of rescuing amphibian diversity.

## Conclusions

The literature describes a variety of disease mitigation strategies that can be applied to amphibians. These include, but are not limited to, reducing host density, treating hosts and habitats, reintroductions with assisted selection, utilizing climatic refugia, immunization, habitat bioaugmentation and host biotherapy, and biocontrol. Beyond maintaining existing biodiversity, many of these strategies aim to reverse the processes of environmental degradation with a restoration ecology approach. By recognizing the opportunistic nature of *Bd *and combining this knowledge with epizootiological models, mitigation strategies can be designed to control disease without the need to completely eliminate the pathogen from the environment.

Conservation biologists should prepare for more active management of amphibian disease on a scale ranging from host to habitat, and varying in approach from medical to ecological. Under development are periodic physical or chemical treatments and methods to limit infectious zoospores in the environment resulting in reduced disease spread or reduced pathogen prevalence or infection intensities. Managing for both abiotic and biotic habitat characteristics may be critical given that healthy microcrustacean communities may actively predate *Bd *zoospores and reduce transmission. Altering amphibian density through culling may not be effective. Refugia where threatened and susceptible species persist must be actively targeted for conservation since they contain the remnants of within-species diversity and potential sources for population recovery, as well as space for potential managed relocation. If recovered or re-colonized populations have evolved increased disease resistance as with other wildlife epizootics [203,204], studying them may reveal new mechanisms for reducing the impact of the disease and suggest strategies for increasing the disease resistance of captive-bred frogs prior to reintroduction. Assisted selection within captive amphibian colonies has long-term potential. Attenuated *Bd *or an avirulent strain could be used as a live vaccine, and perhaps in wild populations after ameliorating the risks of evolving greater virulence. Molecular advances illuminating virulence mechanisms may enable genetic modification of *Bd *and immunogenetics studies may reveal avenues for enhancing immune responses to *Bd *infection [128,205]. Immunization protocols targeting mucosal immunity are needed and could benefit from the process of recovery from an initial infection. Biotherapy has been proven to increase host immunity and may be transmissible within a population or between populations. This hopeful proof-of-concept requires testing on a larger scale. Treatment of individual hosts at the front of an epizootic may slow the spread, allow time for acquired immunity to develop [94], or suppress disease outbreaks through herd immunity. To test these diverse strategies, a step-wise adaptive management approach with continued population and disease monitoring is the best hope for effective disease management. Various combinations of these disease mitigation strategies and creative local solutions are likely to emerge for the stewardship of wild amphibian populations into the future.

## Competing interests

The authors declare that they have no competing interests.

## Authors' contributions

DCW coordinated the review and all authors contributed to drafting and critically revising the manuscript and have read and approved the final version.

## Appendix

Here we provide further details of the ongoing experimental tests of mitigation strategies and of the mathematical model presented in Figure 6. Case studies include: (1) treating individuals, (2) treating pond habitats with fungicides, (3) treating pond habitats by drying, (4) reintroductions with disease monitoring, and (5) biocontrol with microcrustaceans.

### Ongoing case studies

#### 1. Treatment and release (*Alytes obstetricans*, Spain and Switzerland)

In the Peñalara Natural Park (Madrid, central Spain), the first known chytridiomycosis outbreak in Europe rendered the population of *Alytes obstetricans *close to extinction [206]. Tadpole abundance dropped remarkably in successive years (e.g. from more than 5000 to 20 in the pond holding the largest population), considerably increasing the value of each tadpole. Dead or sick adults have never been found in the area, while thousands of dead or dying metamorphs could be easily found. Thus, experimental treatments were restricted to tadpoles.

Pilot tests by J. Bosch (National Museum of Natural Science in Madrid, Spain) verified that tadpoles infected with *Batrachochytrium dendrobatidis *(*Bd*) survived after metamorphosis when kept in captivity at more than 21°C, a temperature higher than ambient field conditions. Therefore, every single tadpole found in the area was collected and kept in the laboratory at high temperature. Metamorphosed animals were then released, even though some of them tested positive for *Bd *by qPCR at the time of release. Prior to release, intensive surveys yielded no metamorphs in the wild. In 2009, the thermal treatment was replaced with itraconazole baths [93], and detailed studies on infection status and survival of released animals are now in progress. Re-infection of treated animals is possible for both kinds of treatments, and it is too early to recommend its use given the risk of *Bd *acquiring resistance to itraconazole.

With the possibility of complete extirpation of *A. obstetricans *in Peñalara Natural Park following outbreaks of chytridiomycosis, a captive-breeding program was established in 2008 by the local government of Madrid, the Museum of Natural History of Madrid (CSIC) and the Durrell Wildlife Conservation Trust. Although the main objective of the program is to maintain a captive breeding colony in case of extinction in the wild, the colony also provides a source for reintroductions. Animals were extremely scarce such that finding founders was difficult. However, because founders were captured from relict metapopulations after 10 years of successive mass mortalities, natural selection has likely occurred. Specifically, it is possible that survivors carry genes fixed by natural selection that confer tolerance (better able to reduce the consequences of infection) rather than resistance (better able to resist *Bd *infection [123]). When offspring are produced, decisions will have to be made about selectively breeding for tolerance or resistance. Presently, we have an incomplete understanding about which components of the host response lead to prevention of infection, elimination of *Bd*, or resolution of disease.

A similar approach is being followed by C. Geiger and B.R. Schmidt (University of Zurich, Switzerland). They collected over-wintered *A. obstetricans *tadpoles from several ponds. The ponds were relatively simple, man-made ponds with amphibian communities consisting of two newt and two anuran species in addition to *Alytes obstetricans*. The tadpoles were taken to the laboratory and treated against *Bd *using itraconazole [93]. *A. obstetricans *tadpoles were selected as a model to test mitigation strategies against *Bd *because they are thought to be a significant *Bd *reservoir. Other amphibian species in the ponds were not treated. Previous mesocosm experiments showed that very few *A. obstetricans *tadpoles treated with itraconazole became re-infected. This was the case even when they were put back into mesocosms where infected conspecifics were present (C. Geiger and B.R. Schmidt, unpublished data).

#### 2. Pond-level treatments - fungicides (Switzerland)

At the University of Zurich, Switzerland, C. Geiger and B.R. Schmidt conducted mesocosm experiments in which they tested whether pond-level treatments against *Bd *are feasible. The use of fungicides is a common method to control fungal pathogens in medicine and agriculture, but Kilpatrick *et al*. [207] described the use of antifungal compounds in natural wetlands to combat *Bd *as "radical". Nevertheless, methods developed in aquaculture may be particularly useful for the development of methods to control *Bd *in natural ponds through the use of fungicides.

Laboratory experiments showed that commonly employed anti-fungal chemicals used in aquaculture and by fish hobbyists can clear *Bd *infection in tadpoles of the midwife toad *Alytes obstetricans *(C. Geiger and B.R. Schmidt, unpublished data). Mesocosm experiments were used to learn whether antifungal chemicals are also effective at eliminating *Bd *from experimental tadpole communities under more natural conditions and how they affect the pond ecosystem. For example, fungi are important decomposers and primary producers in pond food webs [208] and we need to know how the use of fungicides affect ecosystem functions and services. While direct effects of fungicides on pond organisms may be negligible, indirect effects may have strong negative effects [209]. Even if *Bd *could be eliminated from natural environments using fungicides, environmental regulations may prevent the use of fungicides in wetlands.

#### 3. Pond-level treatments - drying (*Alytes muletensis*, Spain)

Drying the habitat containing pathogens can reduce disease incidence. Kriger and Hero [210] showed that *Bd *occurs primarily in permanent ponds but was absent from ephemeral ponds. Thus, draining ponds may be a way to suppress *Bd *in the environment. Because many amphibian species are adapted to ephemeral habitats, draining ponds may not affect amphibian populations negatively [211], especially when done late in the season when tadpoles are no longer present. If the timing of pond drying can be managed, temporary natural or constructed ponds offer a feasible option for managing amphibians impacted by disease. The construction of temporary ponds is already advocated as an amphibian conservation strategy in highly urbanized areas in Europe [212] and is used as a remediation measure in the United States.

Midwife toads (genus *Alytes*) are probably the most *Bd *susceptible species in Europe [35,36,206]. Tadpoles have long lifespans (often more than one year), allowing them to be in permanent contact with zoospores. Adults are highly terrestrial and only males approach the water to release egg clutches. Infected populations of Mallorcan midwife toads, *Alytes muletensis*, seem to be appropriate targets to explore mitigation approaches for several reasons. Populations are contained in a very dry environment which forces animals to move along torrents and impedes migration among different basins [213]. In addition, no other amphibian species co-occur with *A. muletensis*, and pools holding tadpoles are small and relatively free of organic material.

The first attempt to eliminate *Bd *from an *A. muletensis *infected population is in progress [5]. In this attempt, both individuals and the habitat are being treated. The target pool consists of two small cisterns created for watering live-stock in a short torrent gorge. The Mallorcan midwife toad is the only amphibian species inhabiting the pool, and the scarcity of aquatic vegetation, rocks or mud allow the capture of every tadpole. In several visits during a 6-month period, all tadpoles were collected and taken to the laboratory, where they were treated with itraconazole following Garner *et al*. [93]. Field work started before the breeding season with collections of over-wintered tadpoles and continued until no new egg-clutches were found and every tadpole was collected. The pool was completely drained. Treated tadpoles were then put back into the pool after the first autumn rains. We expected that the *Bd *population would not recover once its main tadpole reservoir had been successfully cleared of infection and the ponds dried. However, results from spring of 2010 indicate that reintroduced tadpoles contracted *Bd *infections, but infections were of a lower intensity. Thus, continued eradication efforts will target adults as well as larvae in the area [5].

#### 4. Reintroductions of *Bufo boreas*

Boreal toads have been extirpated from 75% of sites inhabited historically in Rocky Mountain National Park (RMNP) and have declined precipitously in the southern Rocky Mountain Region [214]. In 2007, RMNP launched a thoughtfully planned effort to reintroduce boreal toads. The site was chosen in a region within the park that was historically inhabited by boreal toads [215]. Donor toads were offspring of toads collected in the park and bred in captivity. After three years of surveys, sites were selected based on habitat suitability [216], proximity to existing toad populations, proximity to human activities, logistical considerations, and disease status. Using molecular methods, disease status was determined from skin swabs of boreal chorus frogs, *Pseudacris maculate*, and wood frogs, *Rana sylvatica *[217] and from water samples [218]. *Bd *was not detected at the selected site. Introductions of tadpoles were initially planned for 3 - 5 years. The project has thus far released tadpoles (700 - 14,000) in three consecutive years (2007-2009), and seven adults in 2008. The adults were excess hatchery individuals and released as an opportunity to assess their usefulness as sentinels for disease. These individuals were monitored using radio telemetry and swabbed weekly between June and September. Diagnostic skin swabs revealed the presence of *Bd *in ~30% of sentinels, indicating that *Bd *is still present in the area. In 2009, a handful of one and two year old toads were located at the site. Future releases are planned with extensive monitoring to quantify the success of the reintroduction program.

From this effort, we learned that adults can be effective sentinels for *Bd *presence, and that continued monitoring is extremely important. Monitoring is often the most neglected part of a translocation project [103,110,219] and becomes especially critical when dealing with a transmissible disease and animals that may move relatively long distances. Monitoring is essential and provides information to specify management goals and articulate research hypotheses [220]. As we learn more about the efficacy of mitigation measures, a sound monitoring protocol will be imperative both for disease surveillance and population assessments.

#### 5. Biocontrol with microcrustaceans

The littoral zone of ponds and lakes is a complex area with multiple interactions between biotic and abiotic factors. Especially predator-prey interactions of fish or crayfish and zooplankton, and zooplankton grazing/filter-feeding on smaller microorganisms form a continuous and interdependent cycle over the seasons. The introduction of fish and algae/cyanobacteria into these ecosystems or changes in abiotic factors (pH, chemicals, nutrients, temperature) can destroy established food chains and result in the disappearance or overabundance of some species. The disappearance of cladocerans from Lake Tahoe, CA, in the early 1970's, for example, was linked with high densities of introduced opossum shrimp (*Mysis relicta*) and kokanee salmon (*Onchorynchus nerka*) [221]. A recently study performed by Koksvik *et al*. [222] also correlated a reduction in cladoceran biomass to the introduction of mysids at Lake Jonsvatn, Norway. Whereas cladocerans were highly affected by introduced fish and shrimp, copepods were not or were less negatively impacted (same study).

As filter-feeding organisms, microcrustaceans can be natural predators of *Bd *zoospores which are 3-5 μm in size and contain valuable nutrients, especially lipids [223]. The size of food particles successfully filtered depends on the filter apparatus of the microcrustacean species [224]. Furthermore, some microcrustaceans have food preferences and are able to actively select between different food particles [225]. Kagami *et al*. [185,226] showed that *Daphnia *sp. were feeding on zoospores of the chytrid pathogen (*Zygorhizidium planktonicum*) of the diatom *Asterionella formosa*.

At the Environmental Studies Area (ESA) at California State University Bakersfield, A. Lauer isolated different microcrustaceans (cladocerans, copepods, and ostracods). These were cultured in freshwater supplemented with 'green water', especially *Chlorella *sp. as a food source (L.F.S. Cultures, Oxford, MS). The microcrustaceans were incubated at 15°C in an incubator (Percival E-30B) with a 12 h day (40 μmol quanta m-2s-1) and 12 h night cycle and were growing and multiplying well under these conditions. Cultures of *Bd *(JEL213, obtained from J.E. Longcore, University of Maine) were successfully grown in liquid 1% tryptone medium at 18°C.

In an initial experiment, six *Daphnia *sp. individuals from ESA were incubated together with ~1.5 × 10^5 ^*Bd *zoospores/ml in 5 ml of sterile freshwater (0.22 μm filter sterilized) in wells of a 6-well plate (max. vol. 10 ml) over a period of seven days in comparison to a negative control were no *Daphnia *were added, and a control were only *Daphnia *were present, but no *Bd *zoospores. The experiment was performed in an incubator at 15°C with a day night cycle (12 h under 40 μmol quanta m-2s-1). Prior to the experiment, the *Daphnia *individuals were rinsed three times with sterile freshwater [227] to remove transient microorganisms that could be used as a food source. The same experiment was performed with a local ostracod species (seed shrimp, *Cypridopsis adusta*), isolated from the same environment as *Daphnia *sp. (pond at ESA at CSUB). The species of *Daphnia *used in this experiment still needs to be confirmed. Based on microscopic observations it was most probably *D. rosea *or *D. galeata*.

After the end of the experiment (day 7), all microcrustaceans that were incubated together with *Bd *zoospores were still alive at the end of the experiment, whereas all microcrustaceans without zoospores as a food source died. The well with *Bd *zoospores alone had formed clusters of zoosporangia visible with the naked eye. Microscopic investigations proved that the amount of *Bd *zoospores was under the detection limit in the wells were microcrustaceans had been present. This experiment demonstrated that the two species of microcrustacean tested were actively feeding on *Bd *zoospores. Based on this observation, a second exposure experiment was set up where different amounts of *Daphnia *individuals (0, 4, 8, 12, 16, 20, and 24) were exposed to the same amount of *Bd *zoospores (~1.6 × 10^5 ^spores/ml) over a period of three days in 15 ml of sterile filtered freshwater in sterile collection containers (max. vol. 50 ml) under the conditions described in the pilot experiment. Overall, every 1.5 h a sample of 200 μl was taken which added up to five samples taken each day. The experiment was continued for two more days, taking samples during the day and leaving them untouched during the night. *Bd *zoospores were stained with Malachite green (a spore stain) and counted using a Phase Contrast Microscope (Nikon, type 104) with 400 × magnification and a Neubauer counting chamber.

Even though the initial number of *Bd *zoospores/ml were supposed to be ~1.6 × 10^5^/ml, the numbers varied from 2.3 - 4.2 × 10^5^/ml at the beginning of the experiment. This is probably due to clusters of zoospores that were initially counted as one and then broke apart when the *Bd*-dilutions were prepared. A decline in number of *Bd *zoospores was observed for all trials with a steeper decline when 12 and more *Daphnia *were present in comparison to the control where no *Daphnia *were added to the zoospores (Main text, Figure 5B). After the steep decrease in zoospores on day one of the experiment, the zoospore count stabilized or declined moderately over the next two days (Main text, Figure 5B). It was observed that the *Daphnia *individuals started reproducing in all containers on day 3 of the experiment. Three counts of zoospores were combined and an average calculated for each sample investigated. The exact amount of *Daphnia *individuals at the end of the experiment, their size and biomass was not determined.

In an ongoing project, A. Lauer has begun studying the diversity of microcrustaceans and the presence of *Bd *in different ponds of the Southern San Joaquin Valley and the foothills of the Sierra Nevada (CA), comparing the diversity in the spring and fall. In addition to investigating the microcrustaceans present in the watercolumn, sediment samples were analyzed with molecular methods for the presence of diapausing eggs, which are known to survive for hundreds of years [194]. A discrepancy between the diversity of microcrustaceans in the watercolumn to the one in the sediment might indicate a shift or decline in diversity of microcrustaceans due to environmental influences, such as pollution, eutrophication, invasive species, including the introduction of fish.

## Details of Mathematical Models

### Figure 6*extended legend*

(A) Diagram of the Briggs *et al*. [17] model. The model follows the dynamics of the zoospores (Z) in a zoospore pool, and the sporangia (S_i_) on each frog i. Copied with permission from Proceedings of the National Academy of Sciences. (B) Examples of conservation strategies without an adaptive immune response. Shown here are the fungal loads on a frog in a constant pool of zoospores. In (a), if the frog remains untreated, its fungal load will hit S_max _= 10000 sporangia in 33 days and the frog will die. The default parameters are: ν = 0.25, γ*Z = 500 zoospores/day, η*f = 1 zoospores/sporangia/day, σ = 0.2/day, S(0) = 1 sporangia. In (b)-(d), treatments are applied on day 20. In (b), the zoospore pool is eliminated (γ*Z = 0) on day 20. This extends the life of the frog by slowing the rate of increase of the fungal load on the frog, but for the default parameters, the fungal load continues to build up due to self re-infection. In (c), constitutive defenses of the frog are increased on day 20, perhaps through the application of probiotic bacteria (the fraction of zoospores that successfully infect the frog, ν, is decreased to 0.15 on day 20). This is sufficient to allow the frog to eventually clear the infection. In (d), both types of treatments are applied (on day 20 the frog is removed from the zoospore pool, γ*Z = 0, and exposed to probiotic bacteria that reduce n to 0.15) and the rate of recovery of the frog is increased. (C) Examples of a treatment strategy if the frog has an effective adaptive immune response. The solid red line shows the fungal load on an untreated frog in a constant pool of zoospores. The fungal load builds up rapidly and reaches the lethal threshold on day 33. The dashed blue and dotted green lines show the fungal load and antibody levels if on day 30 the frog is treated with an anti-fungal agent (such as itraconazole), which temporarily reduces its fungal load to zero. The fungal load builds up again, but the initial exposure has resulted in sufficient antibodies that the peak fungal load is below the lethal threshold. The fungal load on the frog eventually reaches a stable level of 916 sporangia. The parameters used are γ*Z = 1 zoospores/day, η*f = 1 zoospores/sporangia/day, σ = 0.2/day, S(0) = 0 sporangia, p = 0.5, and θ = 0.1/sporangia. (D) The parameters used are γ = 1 × 10^-4^/day, η = 9 zoospores/sporangia/day, f = 0.1, σ = 0.2/day, S_i_(0) = 0 sporangia, Z(0) = 10,000 zoospores, p = 0.5, and θ = 0.1/sporangia.

### Model of infection on a single frog without adaptive immunity

The basic Briggs *et al*. [17] model of the interaction between *Bd *and an amphibian host followed the dynamics of the number of sporangia S(t) on each frog in a zoospore pool, Z(t). Here we make the simplifying assumption that a single frog in a large population of frogs will not significantly affect the level of zoospores in the zoospore pool. We instead follow the dynamics of the number of sporangia S(t) on a single frog in an infected population where the density of zoospores is set to a constant value, Z.

The model becomes:

where the parameters are defined as in Briggs *et al*. [17]:

γ = encounter rate between zoospores and frogs in a pool of volume V

ν = fraction of encounters between zoospores and frogs that result in infection of the host (and creation of a new sporangium)

η = release rate of zoospores from each sporangium

f = fraction of released zoospores that immediately infect the same host

σ = loss rate of sporangia from a host (such that 1/σ is the average lifespan of a sporangium)

This can be re-written as:

where b = ν(γ/V) Z is the rate of addition of sporangia to the host from the environment,

and a = (ηνf- σ) is the per-sporangia rate of increase of sporangia in the absence of contributions from the environment.

The solution to this equation, giving the number of sporangia on the frog at any time t, as a function of the initial condition, S(0) = number of sporangia on the frog at time t = 0, is:

The per-sporangia rate of increase of sporangia in the absence of contributions from the environment, a = (ηνf - σ), can be either positive or negative. If a < 0, then the number of zoospores on the frog reaches a stable equilibrium at S* = -b/a, with the frog continually being infected from the zoospore pool. If a>0, then the frog can effectively re-infect itself, and number of zoospores on a frog grows without bounds. If there is a threshold fungal load S_max _above which the frog dies, then with a>0 the sporangia will inevitably surpass this threshold and die due to chytridiomycosis.

### Model of infection on a single frog with adaptive immunity

We developed a model of a hypothetical adaptive immune response to *Bd *infection. It has not yet been demonstrated conclusively that amphibians are able to mount an effective adaptive immune response against *Bd*, although the possibility of such a response remains. To date there have been no published experiments documenting that prior exposure to *Bd *reduces either the susceptibility of frogs to *Bd *infection or the impact of *Bd *on infected hosts (but see [61]). Recent gene expression studies [60,205] have failed to find evidence of up-regulation of genes associated with adaptive immunity in *Bd*-infected frogs compared to uninfected controls, although such studies have been conducted only on *Silurana (Xenopus) tropicalis*, which is highly susceptible to *Bd*. Much more work is needed on adaptive immunity against *Bd*, and several efforts are underway to develop the necessary tools to make this possible (L.A. Rollins-Smith, E.B. Rosenblum pers. comm.). At this point, the model of adaptive immunity presented here is purely speculative.

To the *Model of infection on a single frog without adaptive immunity *described above, we add a second equation that describes the dynamics of a hypothetical mucosal antibody, A, that is produced in response to *Bd *infection. We assume that the rate of production of antibody is proportional to the number of sporangia on the frog (with rate parameter r), and that in the absence *Bd *infection, the level of antibody in the frog would decay exponentially (at rate c). Here we assume that the antibodies are mucosal antibodies that kill off zoospores that encounter the frog skin such that the fraction of zoospores that successfully infect the frog, ν, is no longer a constant but is a function that decreases with increasing antibody level.

The equations describing this model are:

with ν(A) = p*exp(-θ A)

where p = the fraction of encounters between zoospores and frogs that successfully infect the frog when no antibodies are present.

θ = rate at which the fraction of zoospore that successfully infect the frog decreases with increasing antibody titer.

### Population-level model of infection with adaptive immunity

To model a population of frogs with adaptive immunity in a zoospore pool, we follow the dynamics of the number of sporangia S_i _and antibody level A_i _on each frog i. We assume that each frog contributes zoospores to, and becomes infected by, zoospores from a common zoospore pool, Z. We follow the assumption on Briggs *et al*. [17] that a fraction f of the zoospores that are released from each sporangium on frog i immediately re-encounters the same frog (and needs to get past the frog's defenses), with probability ν_i_(A_i_), and the remaining fraction (1-f) enters the zoospore pool.

with ν_i _(A_i_) = p*exp(-θ A_i_) and S_tot _= total number of sporangia on all frogs at time t for all frogs i = 1...N, where N is the current density of frogs.

Frog i dies (and N is decreased by 1) when its fungal load, S_i_, reaches S_max_.

Here we present results of only the deterministic version of the model, in which all frogs with the same initial fungal loads follow identical trajectories.

The model as written does not include birth of frogs, or death due to causes other than chytridiomycosis, and is meant to describe the dynamics within a single year in a frog population in a temperate region. Frog demography can be included in a number of different ways, *e.g*. [17]. This model also includes only a single species of frog. It could be easily expanded to include multiple frog species, each contributing zoospores to separate, overlapping, or a common zoospore pool(s).
